# One-Step Synthesis of Titanium Oxyhydroxy-Fluoride Rods and Research on the Electrochemical Performance for Lithium-ion Batteries and Sodium-ion Batteries

**DOI:** 10.1186/s11671-015-1120-z

**Published:** 2015-10-17

**Authors:** Biao Li, Zhan Gao, Dake Wang, Qiaoyan Hao, Yan Wang, Yongkun Wang, Kaibin Tang

**Affiliations:** Department of Chemistry and Hefei National Laboratory for Physical Science at Microscale, University of Science and Technology of China, Hefei, Anhui 230026 People’s Republic of China

**Keywords:** Lithium-ion batteries, Sodium-ion batteries, Metal oxyhydroxy-fluoride, Hexagonal tungsten bronze, Solvothermal method

## Abstract

Titanium oxyhydroxy-fluoride, TiO_0.9_(OH)_0.9_F_1.2_ · 0.59H_2_O rods with a hexagonal tungsten bronze (HTB) structure, was synthesized via a facile one-step solvothermal method. The structure, morphology, and component of the products were characterized by X-ray powder diffraction (XRD), thermogravimetry (TG), scanning electron microscopy (SEM), transmission electron microscopy (TEM), high-resolution TEM (HRTEM), inductively coupled plasma optical emission spectroscopy (ICP-OES), ion chromatograph, energy-dispersive X-ray (EDX) analyses, and so on. Different rod morphologies which ranged from nanoscale to submicron scale were simply obtained by adjusting reaction conditions. With one-dimension channels for Li/Na intercalation/de-intercalation, the electrochemical performance of titanium oxyhydroxy-fluoride for both lithium-ion batteries (LIBs) and sodium-ion batteries (SIBs) was also studied. Electrochemical tests revealed that, for LIBs, titanium oxyhydroxy-fluoride exhibited a stabilized reversible capacity of 200 mAh g^−1^ at 25 mA g^−1^ up to 120 cycles in the electrode potential range of 3.0–1.2 V and 140 mAh g^−1^ at 250 mA g^−1^ up to 500 cycles, especially; for SIBs, a high capacity of 100 mAh g^−1^ was maintained at 25 mA g^−1^ after 115 cycles in the potential range of 2.9–0.5 V.

## Background

Mixed-anion metal fluorides, which include metal oxyhydroxy-fluorides, metal hydroxy-fluorides, metal oxyfluorides, and so on, have become more and more attractive because of their characteristic structures and properties [1–12]. For example, FeOF_1−x_(OH)_x_ · *n*H_2_O adopted the α-MnO_2_ structure [3], Ti_0.75_(OH)_1.5_F_1.5_ with the ReO_3_-type structure was studied as a UV absorber [8], and Ce_1−x_Ca_x_O_2−x−y/2_F_y_ with the fluorite-type structure exhibited UV-shielding property [10]. In recent years, to extend the scope of existing electrode materials for lithium-ion batteries (LIBs) as well as sodium-ion batteries (SIBs) and make more progress in the development of clean energy, the use of mixed-anion metal fluorides was more and more popular [5, 13–24]. Iron-based fluorides with the hexagonal tungsten bronze (HTB) structure were regarded as potential candidates for cathode material, and the research on their electrochemical performance has been ongoing for a period of time [5, 13]. Demourgues et al. reported that FeF_2.2_(OH)_0.8−y_O_y/2_□_y/2_ with the HTB structure displayed better cyclability and reversible capacity than the precursor FeF_2.2_(OH)_0.8_ · (H_2_O)_0.33_ for LIBs [5]. Wang et al. reported that FeF_3_ · xH_2_O including the HTB structure FeF_3_ · 0.33H_2_O exhibited a discharge capacity of 101 mAh g^−1^ after 30 cycles in the potential range of 4.0–1.0 V at 0.1 C, which was quite high for SIBs [13]. Importantly, the electrochemical characteristics of the original samples proved the potentiality for further research.

In fact, another metal oxyhydroxy-fluoride with the HTB structure, titanium oxyhydroxy-fluoride has been reported by Demourgues et al. [2]. With the same one-dimension channels in structure and lighter Ti atomic weight, titanium oxyhydroxy-fluoride is also considered to be an attractive electrode material which supports Li/Na intercalation/de-intercalation into the host structure. The electrochemical reaction is supposed to benefit keeping certain electrode potential and studying how the HTB structure influences the electrochemical properties.

Herein, we demonstrate a facile one-step method to synthesize titanium oxyhydroxy-fluoride rods by introducing anions into titanium binary fluoride. The synthesis route started with the use of TiF_4_ powder (fluorine source), ethanol, and deionized water. The deionized water and ethanol volume using in reaction played significant roles in controlling the structure and morphology of the products, respectively. Structure features were studied by analyses of X-ray powder diffraction (XRD), thermogravimetry (TG), high-resolution TEM (HRTEM), and so on. Besides, the electrochemical properties of as-prepared half-cells were evaluated in detail for both LIBs and SIBs, which may help shed new light on the Li/Na intercalation/de-intercalation mechanism.

## Methods

### Preparation

Titanium oxyhydroxy-fluoride was prepared as follows: TiF_4_ (Alfa Aesar 98 %) was firstly ground to fine powder in a glove box. After that, 1 g TiF_4_ was taken out and put in a 10 mL Teflon autoclave, then 1.20 mL deionized water and a certain volume of ethanol were put in the autoclave sequentially. Operation in this step should be quick to avoid earlier hydrolysis of TiF_4_. The autoclave was heated to 150 °C and kept for 48 h. The precipitate was centrifuged, washed with deionized water over three times, and kept in a vacuum oven at 60 °C for 12 h to get the final products. The ethanol volume used can be described as follows: 1.88 mL ethanol for long titanium oxyhydroxy-fluoride rods, 0.47 mL ethanol for short titanium oxyhydroxy-fluoride rods, and 0.94 mL ethanol for titanium oxyhydroxy-fluoride with the morphology of hexagonal rods. In this article, the sample used for TG and galvanostatic charge–discharge tests is long TiO_0.9_(OH)_0.9_F_1.2_ · 0.59H_2_O rods.

### Characterization

The crystal structure features of as-prepared materials were characterized by X-ray powder diffraction on a Philips X’pert X-ray diffractometer using Cu Kα1 radiation at room temperature. The morphology images of as-prepared materials were taken on a JEOL JSM-6700F scanning electron microscope. The transmission electron microscopy (TEM) and HRTEM images were taken on a JEOL-2010 transmission electron microscope. The thermostabilization of as-prepared materials was characterized by thermogravimetry analysis on a DTG-60H thermogravimetry analyzer which worked under nitrogen atmosphere at a heating rate of 10 °C/min from room temperature to 700 °C. The Ti content was characterized by inductively coupled plasma optical emission spectroscopy (ICP-OES) using Atomscan Advantage. The F content was characterized by ion chromatograph (ICS-3000). The [F]/[Ti] molar ratio was further confirmed by energy-dispersive X-ray (EDX) analysis recorded on the JEOL-2010 transmission electron microscope.

### Electrochemical Measurement

The electrochemical behavior of all the aforementioned products was tested for LIBs and SIBs by the galvanostatic charge–discharge method at room temperature. For LIBs, the half-cells were composed of the as-prepared product as working electrode, Li metal as counter and reference electrode, and the 1 M LiPF_6_ ethylene carbonate/dimethylcarbonate (volume ratio 1:1) solution as electrolyte, which were assembled as coin cells (2016 R-type). The composition of the working electrode was active material (70 %), Super P-carbon black (20 %), and sodium carboxymethyl cellulose binder (10 %); 70, 20 and 10 % were the weight percentage. The above mixture was ground in water and then homogeneously pasted on a Cu slice; after that, the Cu slice was dried in a vacuum oven which was heated to 80 °C for 12 h. The assembling was operated in a glove box. The glove box was full of argon, in which oxygen and H_2_O were kept under 0.1 ppm. For SIBs, the preparation of Cu slice with the active material was in the same procedure which has been introduced above. The differences were we used Na metal as counter and reference electrode, fiberglass instead of polypropylene as separator, and 1 M NaClO_4_ in propylene carbonate as electrolyte. Tests of electrochemical performance were taken on a Neware-BTS-TC53 instrument at required current densities and in the appropriate electrode potential window.

## Results and Discussion

**Fig. 1:**
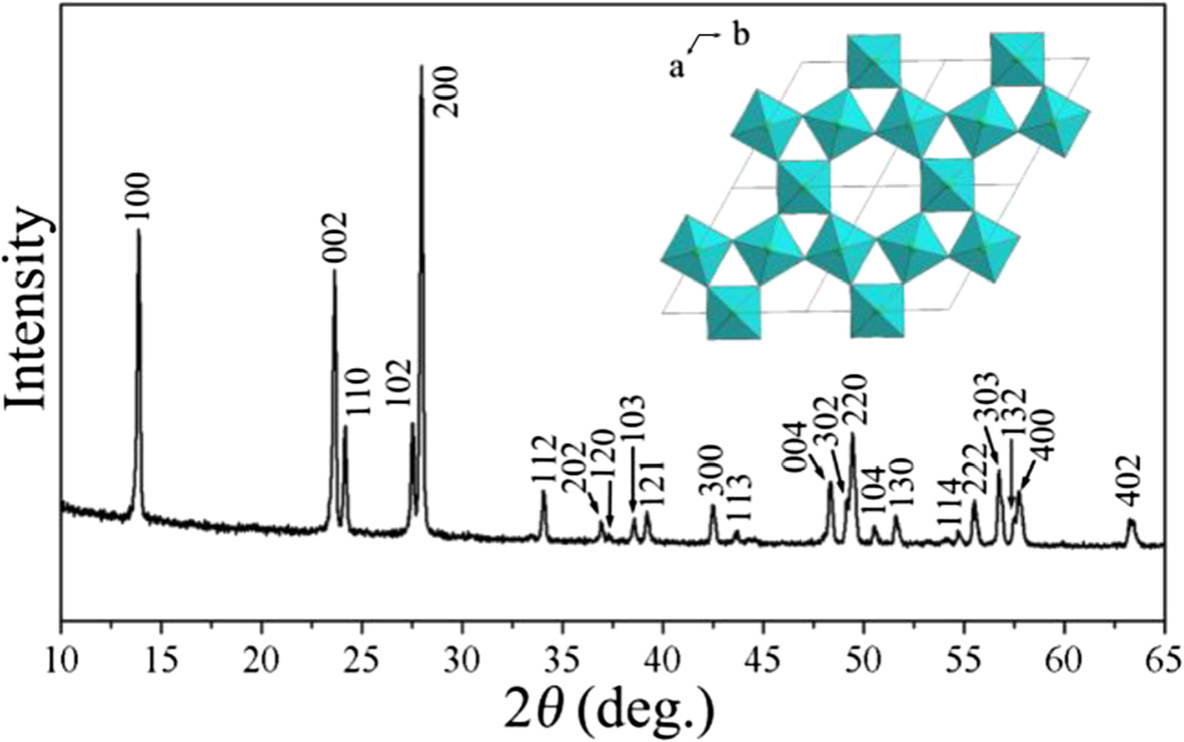
XRD pattern of titanium oxyhydroxy-fluoride. The HTB structural frame is shown as inlet

The XRD pattern of titanium oxyhydroxy-fluoride is shown in Fig. 1. All of the diffraction peaks are indexed by a hexagonal lattice with the lattice parameters *a* = 7.3636 Å, *c* = 7.5186 Å, and the space group P6_3_/mmc, which are consistent with the HTB-Ti_0.75_O_0.25_(OH)_1.3_F_1.2_ reported by Demourgues et al. [2]. It is confirmed that titanium oxyhydroxy-fluoride had the HTB structure without observed impurity. The atom mole ratio [F]/[Ti] is 1.2 which is confirmed by analyzing the results of ion chromatograph, ICP-OES, and EDX. The HTB structural frame shown as inlet is viewed down the [001] direction; the one-dimension channels in the structure are expected to benefit Li and Na intercalation/de-intercalation. It is noteworthy that, the XRD patterns of titanium oxyhydroxy-fluoride with different morphologies are identical.

**Fig. 2:**
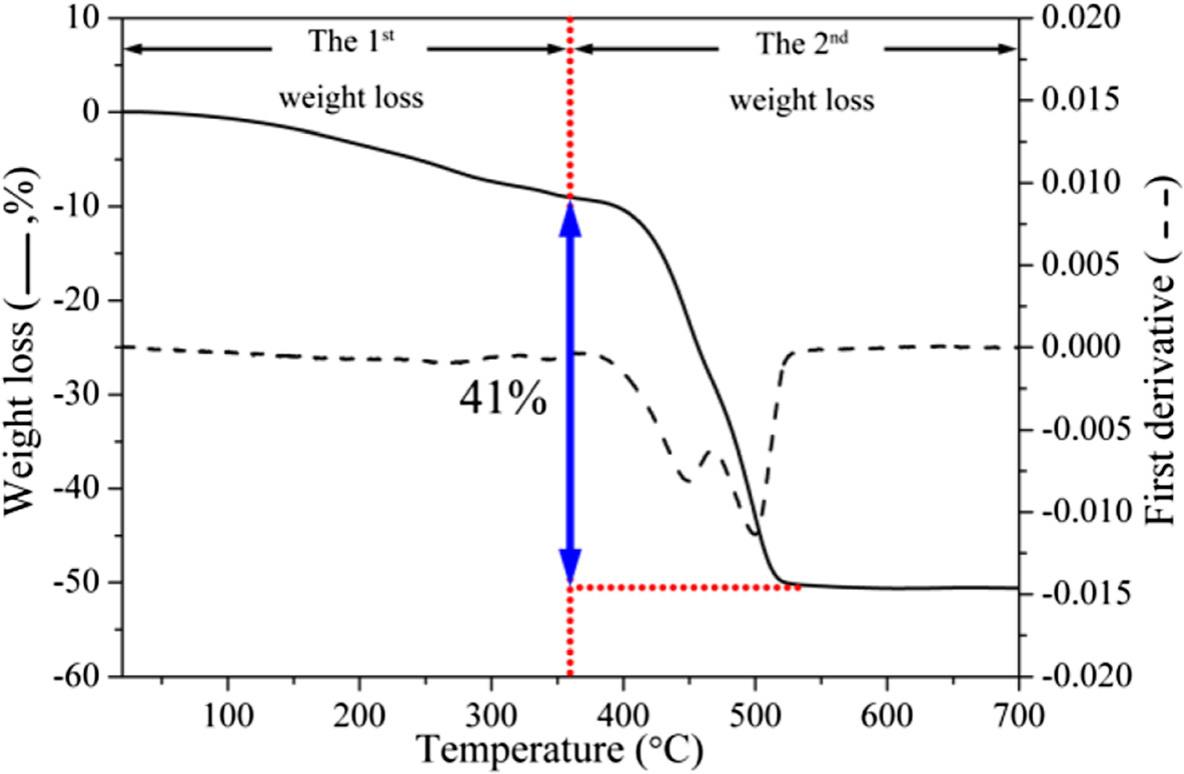
TG curves of titanium oxyhydroxy-fluoride

TG curves of titanium oxyhydroxy-fluoride are shown in Fig. 2. There are two weight losses in the thermal decomposition of titanium oxyhydroxy-fluoride from room temperature to 700 °C. Firstly, a weight loss of approx 9 % occurs and ends at 370 °C. It is considered that the first weight loss is related to the water absorbed on titanium oxyhydroxy-fluoride powders. Secondly, a bulky weight loss of 41 % occurring at 370–520 °C is considered to be due to the thermal decomposition of titanium oxyhydroxy-fluoride. In this period, OH and F constituents depart in the form of H_2_O and TiF_4_ (g), respectively. It can also be considered that the sequential H_2_O departure and TiF_4_ loss correspond to the two peaks of the first derivative, for the fact under certain temperature only H_2_O departure is observed in the thermal treatment of FeF_2.2_(OH)_0.8_ · (H_2_O)_0.33_ which is reported by Demourgues et al. [5]. The thermal decomposition of titanium oxyhydroxy-fluoride finally leads to the formation of TiO_2_ when the temperature reaches 520 °C. The bulky weight loss and decomposition temperature of titanium oxyhydroxy-fluoride are similar to TiOF_2_ which has been studied recently [16, 25]. After taking into consideration of the analyses of the TG curves and the mole ratio [F]/[Ti], the formula for titanium oxyhydroxy-fluoride should be proposed as: TiO_0.9_(OH)_0.9_F_1.2_ · 0.59H_2_O, which is analogous to the one presented by Demourgues et al. [2].

**Fig. 3:**
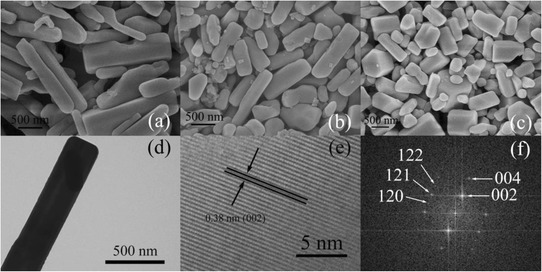
SEM images of titanium oxyhydroxy-fluoride, TiO_0.9_(OH)_0.9_F_1.2_ · 0.59H_2_O with different morphologies: long rods (**a**), short rods (**b**) and hexagonal rods (**c**); TEM image (**d**), HRTEM image (**e**) and the corresponding FFT image (**f**) of long TiO_0.9_(OH)_0.9_F_1.2_ · 0.59H_2_O rods; the arrows in the HRTEM image (**e**) indicate the 0.38 nm interfringe spacing, and the arrows in the corresponding FFT image (**f**) indicate the spots which represent different lattice planes of the product

The morphology and structure features of titanium oxyhydroxy-fluoride, TiO_0.9_(OH)_0.9_F_1.2_ · 0.59H_2_O particles are also investigated through the analyses of scanning electron microscopy (SEM), TEM, HRTEM images and the corresponding FFT image. The size of long TiO_0.9_(OH)_0.9_F_1.2_ · 0.59H_2_O rods (Fig. 3a) is approx 2 μm long and 100–500 nm in diameter; the typical long rod with the regular shape is shown in Fig. 3d. For short TiO_0.9_(OH)_0.9_F_1.2_ · 0.59H_2_O rods (Fig. 3b), the length ranges from 700 to 1300 nm and the diameter ranges from 250 to 400 nm. All the TiO_0.9_(OH)_0.9_F_1.2_ · 0.59H_2_O rods with different morphologies are synthesized under the same condition with the only difference of the ethanol volume. Actually, if only the volume of deionized water is changed (from 1.20 mL to 0.15 mL), another compound hexagonal TiOF_2_ is synthesized. The sole difference between the synthesis of TiO_0.9_(OH)_0.9_F_1.2_ · 0.59H_2_O and hexagonal TiOF_2_ ,i.e., the volume of deionized water indicates that during the synthesis of TiO_0.9_(OH)_0.9_F_1.2_ · 0.59H_2_O, more hydrolysis reactions of TiF_4_ occur and Ti^4+^ binds the generated hydroxy to form the final product with the HTB structure. In other words, the hydroxy groups are considered to be very important for the firm HTB structure. It can also be concluded that the deionized water volume of reaction decides the structure of the products while the ethanol volume plays a significant role in affecting the morphology. The 0.38-nm interfringe spacing in Fig. 3e corresponds to the (002) lattice plane of TiO_0.9_(OH)_0.9_F_1.2_ · 0.59H_2_O. The vertical (120) and (002) lattice plane in Fig. 3e are also confirmed by the FFT image (Fig. 3f).

**Fig. 4:**
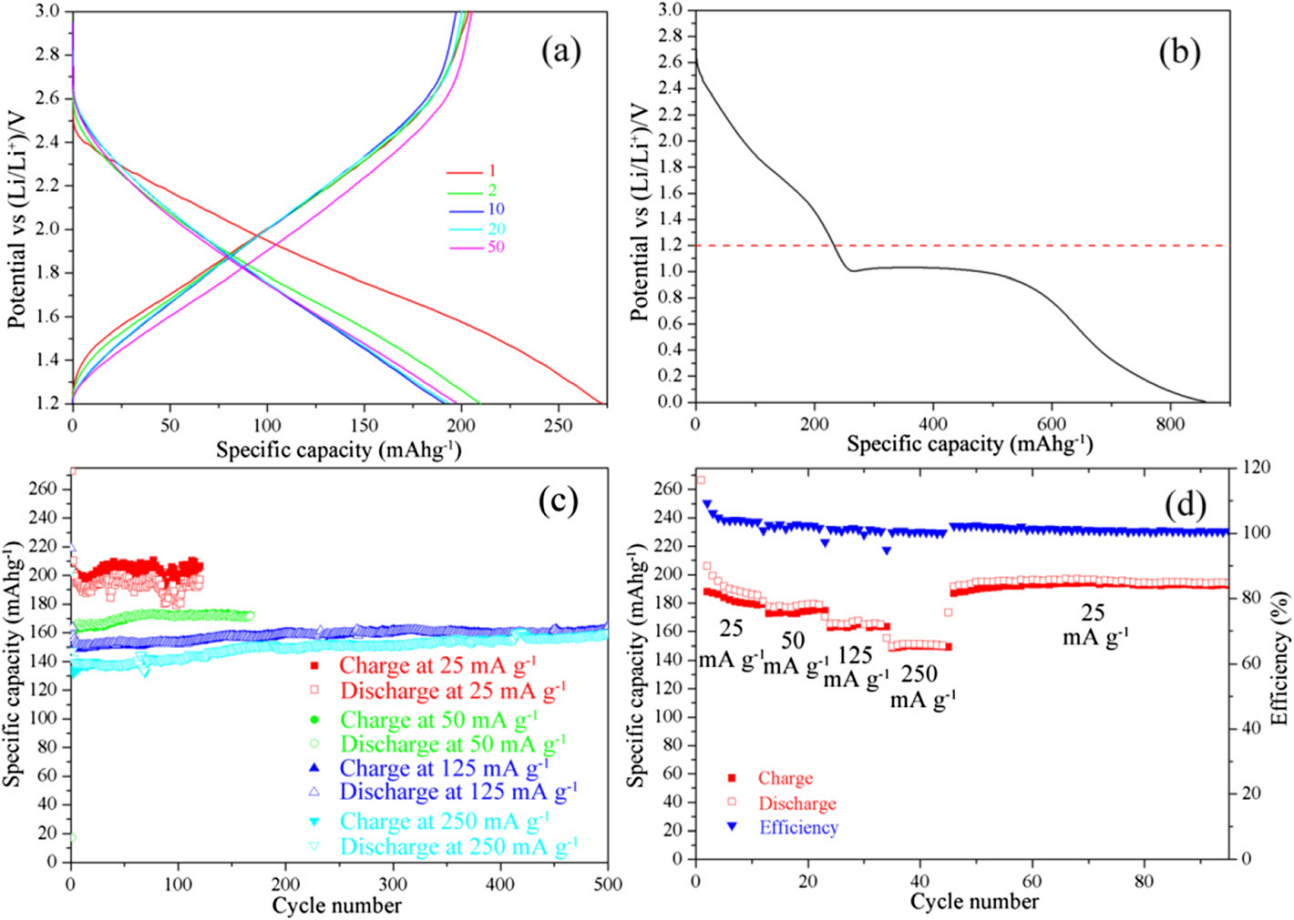
**a** Charge and discharge curves of TiO_0.9_(OH)_0.9_F_1.2_ · 0.59H_2_O for LIBs, the half-cell is performed at 25 mA g^−1^; several selected cycles are shown for clarity; **b** first discharge curve of TiO_0.9_(OH)_0.9_F_1.2_ · 0.59H_2_O which is performed at 25 mA g^−1^ in the potential range of 3.0–0.05 V; **c** cycling performance of TiO_0.9_(OH)_0.9_F_1.2_ · 0.59H_2_O; and **d** rate capacity of one TiO_0.9_(OH)_0.9_F_1.2_ · 0.59H_2_O half-cell for LIBs between 3.0–1.2 V, different current densities are labeled

**Fig. 5:**
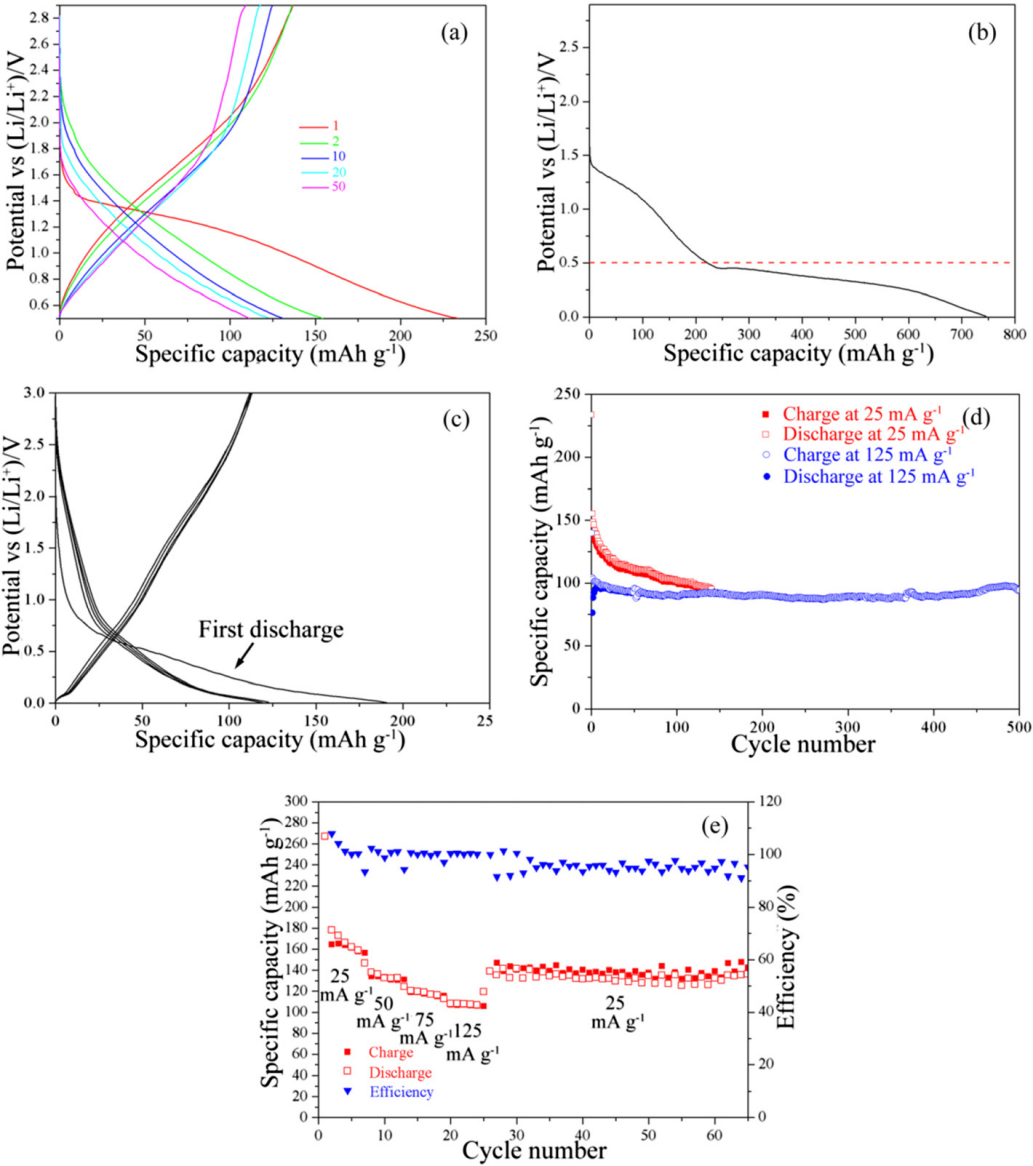
**a** Charge and discharge curves of TiO_0.9_(OH)_0.9_F_1.2_ · 0.59H_2_O for SIBs in the potential range of 2.9–0.5 V; several selected cycles are shown for clarity; **b** first discharge curve of TiO_0.9_(OH)_0.9_F_1.2_ · 0.59H_2_O in the potential range of 2.9–0.05 V; **c** the former 5 cycles of hexagonal TiOF_2_ half-cell for SIBs; all the half-cells are performed at 25 mA g^−1^; **d** cycling performance of TiO_0.9_(OH)_0.9_F_1.2_ · 0.59H_2_O for SIBs; and **e** rate capacity of one TiO_0.9_(OH)_0.9_F_1.2_ · 0.59H_2_O half-cell for SIBs between 2.9 and 0.5 V, different current densities are labeled

To investigate the electrochemical properties of titanium oxyhydroxy-fluoride TiO_0.9_(OH)_0.9_F_1.2_ · 0.59H_2_O, both LIBs and SIBs are performed by using galvanostatic charge/discharge method. The results are shown in Figs. 4 and 5. For LIBs, during the first cycle, the discharge plateau appears at 2.4 V and last until the electrode potential reaches 1.2 V, and the only one plateau suggests that there is only one lithiated TiO_0.9_(OH)_0.9_F_1.2_ · 0.59H_2_O phase ,i.e., no other phase transition of TiO_0.9_(OH)_0.9_F_1.2_ · 0.59H_2_O is observed during the lithiation of TiO_0.9_(OH)_0.9_F_1.2_ · 0.59H_2_O. The potential range where Li intercalation/de-intercalation occurs in is 3.0–1.2 V, which can also be illustrated by Fig. 4b. The specific capacity of the first discharge and the first charge is 270 mAh g^−1^ and 200 mAh g^−1^, while the specific capacities of the following cycles, whether of charge or of discharge, are approx 200 mAh g^−1^. TiO_0.9_(OH)_0.9_F_1.2_ · 0.59H_2_O exhibits stabilized 200, 170, 150, and 140 mAh g^−1^ at 25, 50, 125, and 250 mA g^−1^, respectively, in the electrode potential range of 3–1.2 V without observed fading for 120–500 cycles (Fig. 4c and d), which is pretty satisfying and illustrates the highly reversible nature. Besides, after cycling at 25 mA g^−1^ again, the specific capacity of TiO_0.9_(OH)_0.9_F_1.2_ · 0.59H_2_O is recovered (Fig. 4d). It is noteworthy that the stabilized 200 mAh g^−1^ capacity of TiO_0.9_(OH)_0.9_F_1.2_ · 0.59H_2_O is quite large, compared with that of Li_4_Ti_5_O_12_ (theoretical capacity 170 mAh g^−1^). The good rate capacity and cycling stability are connected with the one-dimension channels in the HTB structure and the morphology of homogeneous rods.

For SIBs, in the first cycle the discharge plateau appears at about 1.6 V and ends at 0.5 V which is higher than TiO_2_ [26], and the potential of Na intercalation/de-intercalation is above 0.5 V which is shown in Fig. 5b. The specific capacity of the first discharge is 233 mAh g^−1^ (Fig. 5a), and after the first discharge, the specific capacity gradually decreases from 150 mAh g^−1^ (the second discharge) to 111 mAh g^−1^ (the 50th discharge), but it is still above 100 mAh g^−1^ after 115 cycles (Fig. 5d). The electrochemical performance of another product obtained under the similar reaction condition, hexagonal TiOF_2_, is shown in Fig. 5c, the details of this work is introduced in another article which is submitting. The capacities of the former 20 cycles of the TiO_0.9_(OH)_0.9_F_1.2_ · 0.59H_2_O half-cell in the potential range of 2.9–0.5 V are higher than hexagonal TiOF_2_ even the potential ranges from 3.0 to 0.05 V, which is considered to be facilitated by the HTB structure. The TiO_0.9_(OH)_0.9_F_1.2_ · 0.59H_2_O half-cell cycled at 125 mA g^−1^ stabilizes at a specific capacity of 100 mAh g^−1^ and it is considered to be due to the incomplete Na intercalation/de-intercalation (Fig. 5d). The rate capacity of TiO_0.9_(OH)_0.9_F_1.2_ · 0.59H_2_O for SIBs is shown in Fig. 5e, TiO_0.9_(OH)_0.9_F_1.2_ · 0.59H_2_O exhibits a capacity of 160, 130, 120, and 100 mAh g^−1^ cycled at 25, 50, 75, and 125 mA g^−1^, respectively. 76 % capacity of the second discharge ,i.e., 130 mAh g^−1^ is retained after cycling at 25 mA g^−1^ again without observed fading for another 35 cycles. Compared with LIBs, the electrochemical performance of TiO_0.9_(OH)_0.9_F_1.2_ · 0.59H_2_O for SIBs is restricted, whether the capacities or the cycling stability, which may be related to the different ion sizes and diffusion rates of Li and Na ions.

## Conclusions

In summary, titanium oxyhydroxy-fluoride TiO_0.9_(OH)_0.9_F_1.2_ · 0.59H_2_O with the HTB structure is synthesized by a facile one-step method. The purity of the products is confirmed, and products with various morphologies are obtained by adjusting the volume of ethanol used in the synthesis process. The electrochemical performance of TiO_0.9_(OH)_0.9_F_1.2_ · 0.59H_2_O is also studied to investigate Li/Na intercalation/de-intercalation in the HTB structure. For LIBs, TiO_0.9_(OH)_0.9_F_1.2_ · 0.59H_2_O delivered a stabilized capacity of 200 mAh g^−1^ under a current density of 25 mA g^−1^ in the former 120 cycles; the capacities of 150 mAh g^−1^ and 140 mAh g^−1^ are also exhibited without observed fading after 500 cycles at 125 mA g^−1^ and 250 mA g^−1^, respectively, which demonstrated the highly reversible cycling stability of TiO_0.9_(OH)_0.9_F_1.2_ · 0.59H_2_O. For SIBs, TiO_0.9_(OH)_0.9_F_1.2_ · 0.59H_2_O exhibits capacities above 130 mAh g^−1^ in the former 20 cycles and capacities above 100 mAh g^−1^ in the former 115 cycles, which is quite large and it is potential for the further improvement of TiO_0.9_(OH)_0.9_F_1.2_ · 0.59H_2_O. We believe TiO_0.9_(OH)_0.9_F_1.2_ · 0.59H_2_O is potential as an electrode material, and the electrochemical properties will be improved by further research such as nanocomposite and carbon-coated process.
